# Collaborative Integration of Community Health Workers in Hospitals and Health Centers to Reduce Pediatric Asthma Disparities: A Quality Improvement Program Evaluation

**DOI:** 10.1007/s10900-024-01331-y

**Published:** 2024-02-23

**Authors:** Sweta Basnet, Kristen Wroblewski, Elizabeth Hansen, Ernestina Perez, Ruobing Lyu, Zain Abid, Alexis Roach, Catina Latham, Nadia Salibi, Brenda Battle, Louise Giles

**Affiliations:** 1 Urban Health Initiative, University of Chicago Medical Center, 5841 S. Maryland Ave, Chicago, IL 60637 USA; 2 Department of Public Health Sciences, The University of Chicago, Chicago, IL USA; 3 Harris School of Public Policy, The University of Chicago, Chicago, IL USA; 4 Department of Pediatric Medicine, The University of Chicago, Chicago, IL USA

**Keywords:** Pediatric Asthma, Community Health Worker, Quality Improvement, Emergency Department Visits, Health Disparities

## Abstract

To address pediatric asthma disparities on the South Side of Chicago, a community health worker (CHW) home visiting intervention was implemented collaboratively by academic institutions and community based health centers. This evaluation assessed the effectiveness of this longitudinal quality improvement CHW intervention in reducing asthma morbidity and healthcare utilization. All patients aged 2–18 who met the high-risk clinical criteria in outpatient settings or those who visited the ED due to asthma were offered the program. A within-subject study design analyzed asthma morbidity and healthcare utilization at baseline and follow-up. Multivariable mixed-effects regression models, adjusted for baseline demographic and asthma characteristics, were used to assess changes over time. Among 123 patients, the average age was 8.8 (4.4) years, and 89.3% were non-Hispanic black. Significant reductions were observed in the average daytime symptoms days (baseline 4.1 days and follow-up 1.6 days), night-time symptoms days (3.0 days and 1.2 days), and days requiring rescue medication (4.1 days and 1.6 days) in the past two weeks (all *p* < 0.001). The average number of emergency department visits decreased from 0.92 one year before to 0.44 one year after program participation, a 52% reduction (*p* < 0.001). No significant difference was found in hospital admissions. These results support the use of a collaborative approach to implement the CHW home visiting program as part of standard care for pediatric asthma patients in urban settings. This approach has the potential to reduce asthma disparities and underscores the valuable role of CHWs within the clinical care team.

## Introduction

Over 4.6 million children in the US have asthma, with higher prevalence among Black children (11.6%) compared to White children (5.5%) [1]. The economic burden of pediatric asthma is significant. A recent literature review estimated that the total direct cost of pediatric asthma to the US was $5.92 billion [2]. The South Side of Chicago has some of the worst economic, health, social, and violence outcomes in the U.S. Included in this is the University of Chicago Medicine (UCM) 12 zip-code service area, which is home to over 600,000 residents, the majority of whom are historically marginalized and people of color (73.7% African-American, 14.6% Hispanic or Latino) [3]. Due to generations of structural inequality and systemic disinvestment in social, economic, and health-promoting infrastructure, residents on the South Side also suffer significantly higher rates of chronic health conditions, one of the primary drivers of the life expectancy gap between Blacks and non-Blacks [4].

According to IHA COMPData, the rate of asthma-related emergency department (ED) visits in UCM’s service area among pediatric patients aged 5–17 years is nearly 3 times greater than that in Illinois (1,140 visits per 100,000 residents versus 396 visits per 100,000 residents) [5]. The Community Health Needs Assessment conducted by UChicago Medicine in 2015 placed pediatric asthma as a top health priority [6]. Addressing pediatric asthma is difficult due to limited access to healthcare on the South Side of Chicago, lack of resources needed to address wide-ranging social determinants of health (“SDOH”), and the general hardships associated with navigating a fragmented health care delivery system.

The Community Health Worker (CHW) model uses layperson-level public health workers who serve as liaisons between patients and the health care system, and are trained to assist in providing patients with linkages to care and managing chronic health conditions such as asthma [7]. Previous studies utilizing the home-based CHW model across urban areas of the United States have shown reductions in home environmental triggers, asthma-related symptoms, missed school days, and healthcare utilization [8–16]. Much of the existing evidence is based on research studies with rigid follow-up periods, specific populations (for example, Medicaid-enrolled only) or limited intervention scopes (focusing only on environmental triggers vs. a more holistic approach). In 2008, the Community Preventive Services Task Force (an initiative of the U.S. Department of Health and Human Services) recommended a home-based community health worker intervention for children with asthma [17]. However, healthcare systems have been slow to adopt home-based interventions for asthma as a standard practice, and limited evidence exists for real-life implementation and effectiveness of CHW asthma interventions *within* the U.S. healthcare system. The well-known Community Asthma Initiative from Boston is a quality improvement program that is implemented as part of the healthcare system and has continuously demonstrated the effectiveness of the home-based CHW model in reducing asthma morbidity, health-care utilization and costs [18–21].

UChicago Medicine introduced its own CHW program to address pediatric asthma disparities in 2016 as a partnership of healthcare organizations that includes UChicago Medicine Comer Children’s Hospital, La Rabida Children’s Hospital, Friend Family Health Center, St. Bernard Hospital, Beloved Community Family Wellness Center and Chicago Family Wellness Center. Coined South Side Pediatric Asthma Center (“SSPAC”), this program employs community members trained as CHWs to work with patients and their families to better understand and manage their child’s asthma [22]. CHWs provide health education, conduct home environmental assessments to identify asthma triggers and provide remediation supplies, communicate with healthcare team members, and connect people to resources to address social determinants of health. The CHW program was modeled after the Community Asthma Initiative from Boston and the Sinai Urban Health Institute’s CHW model, and both groups provided technical assistance during the program design phase [13, 21].

SSPAC was implemented to reduce asthma disparities on the South Side of Chicago through home visits and comprehensive asthma management and follow-up. This study evaluated data from patients participating in SSPAC’s CHW program at multiple healthcare sites to assess the program’s effectiveness in reducing asthma morbidity and improving health care utilization outcomes when CHWs were incorporated into the continuum of care.

## Methods

### Intervention

Since 2016, the SSPAC program has integrated 1 CHW within each of the 4 SSPAC member specialty care clinics (St. Bernard Hospital, La Rabida Children’s Hospital, Friend Family Health Center, and Comer Children’s Hospital at the University of Chicago). CHWs were available to provide education to patients and caregivers after doctor visits, and were referred to patients who needed follow-up home visits.

All patients aged 2–18 years who met the high-risk clinical criteria set for the program and were verified by the clinician were approached for the program. High-risk included meeting any one of the following criteria: 8 or more days of daytime symptoms in the past month, 8 or more days of rescue medication usage in the past month, 2 or more days of nighttime symptoms in the past month, any hospitalization or ED visits in the past 6 months, or ever been admitted to the ICU for asthma. The CHW manager and CHW supervisor at the UChicago Medicine site reviewed the comprehensive patient list from all participating sites (including both emergency departments and outpatient clinic sites) to identify and enroll patients in the program. All CHWs attempted to see eligible patients in the ED or in the clinic to enroll them for home visits. For patients whom CHWs were unable to see in the ED or in the clinic, CHWs called them within 48 h of discharge to attempt program enrollment. Patients who did not live in the UCM 12-zipcode service area were not eligible for the program.

The program was initially designed to engage patients for 6 months to 1 year through 1–8 home or phone visits. However, based on learning from CHWs and process data, the program went through continuous iterations to be flexible on the number of visits and duration of participation to meet the needs of each family. During these visits, the CHWs provided comprehensive education on asthma management, conducted home environmental assessments and trigger remediation, and assisted with patient care coordination. Asthma management education included the following topics: signs and symptoms of asthma, types of medications and their usage, inhaler technique, and recognition of environmental triggers with suggestions for their removal and management in the home. Consent was obtained at the first visit to use the data for evaluation purposes and to communicate with the child’s healthcare providers across multiple partner clinics. The majority of home visits occurred in the child’s home; however, for those parents who did not want to meet CHWs in their home, accommodations were made to meet in public spaces. During each home or phone visit a standard assessment was completed investigating the child’s asthma symptoms, medication adherence, health care utilization, and environmental trigger exposure.

After the first patient visit and assessment, the CHWs maintained communication with the child’s primary care provider and/or asthma care provider on the basis of the status of the child’s asthma and observations from home visits. The CHWs ensured that all enrolled children had follow-up appointments with their asthma provider and accompanied their patients to appointments (when possible) to continue rapport building with patients and healthcare providers.

### Data Collection

Study data were collected and managed using REDCap electronic data capture tools hosted by UChicago Medicine. Research Electronic Data Capture (REDCap) is a secure, web-based software platform designed to support data capture for research studies, providing (1) an intuitive interface for validated data capture; (2) audit trails for tracking data manipulation and export procedures; (3) automated export procedures for seamless data downloads to common statistical packages; and (4) procedures for data integration and interoperability with external sources [23, 24].

All the data were self-reported by the caregivers and subsequently collected and entered into REDCap by the CHWs. The data were tracked over the course of the program for all the children across all the SSPAC member sites. Data from participants who were enrolled in the program and who received visits from May 1, 2016 to December 31, 2018, were included in the evaluation to ensure that at least one year of follow-up data was available. This project received a formal Determination of Quality Improvement status from The Center for Healthcare Delivery Science and Innovation at University of Chicago Medicine. As such, this initiative was deemed not human subject research and therefore not reviewed by the Institutional Review Board.

### Measures

#### Asthma Morbidity

Self-reported data were collected at baseline visits and at every subsequent phone or home visit. The short-term outcomes of interest were the frequency of daytime asthma symptoms, nighttime asthma symptoms, rescue medication use, and activity limitation. All short-term outcomes of interest were measured using a recall period of the past 2 weeks.

A categorical variable for asthma control at baseline (well controlled, partly controlled, and uncontrolled) was generated by adapting the guidelines set forth by the Global Initiative for Asthma 2018 [25]. The program assessments asked, in the past two weeks did the child have:

- Daytime symptoms ≥ 4 days.
- Rescue medication use ≥ 4 days.
- Night symptoms ≥ 1.
- Activity limitation ≥ 1.

“Well controlled” was defined as those who had none of the above (daytime symptoms less than 4 days, rescue medication use less than 4 days, no night symptoms, and no activity limitations). “Partly controlled” were those who had one to two of the criteria. “Uncontrolled” were those who had three to four of the criteria.

The median household income at the zip code level was extracted from the U.S Census Bureau American Community Survey (ACS) 2019 data and matched with program data using the home zip codes of participants [26]. To adjust for seasonality, a variable (spring, summer, fall, and winter) was created based on the month of the first visit.

#### Healthcare Utilization and Missed School and Work Days

The long-term outcomes of interest were: asthma-related emergency department visits, missed school days since the last CHW visit, and missed work days since the last CHW visit. All long-term outcomes of interest were measured using a recall period of “past 6 months” at baseline with the exception of asthma-related hospitalizations. Asthma-related hospitalization data were collected using a recall period of 1 year prior to the baseline date. At post- baseline encounters, all long-term outcomes of interest were measured from prior CHW visits (i.e., “since last visit”).

#### Environmental Triggers

Exposure (present vs. not present) to environmental triggers (tobacco smoke, pets, mold, rat, cockroaches, and dust on the child’s sleeping surface) was self-reported and observed during the second and subsequent visits.

#### Covariates

The demographic and asthma characteristics included: age, gender, race/ethnicity (non-Hispanic Black vs. Other), use and availability of controller medication (has and uses all 14 days, has and uses less than 14 days, and does not have), and proxy for asthma severity (history of ICU admission); all the covariates were self-reported at baseline. Knowledge scores were based on the knowledge assessment completed by the parents which assessed their knowledge about the types of asthma medications, when to use them and what actions to take during asthma attacks.

### Analysis

A multivariable logistic regression model was used to assess factors associated with dropout after the first visit. The covariates included baseline demographic and asthma characteristics. For short-term outcome variables (i.e., frequency of daytime asthma symptoms, nighttime asthma symptoms, rescue medication use, and activity limitation), reported days at baseline and median days reported over the follow-up period were initially compared using paired t-tests. Then, multivariable mixed-effects negative binomial regression models were fit to assess the changes over time and adjusted for baseline demographic and asthma characteristics.

For self-reported and hospital administrative data for asthma morbidity and healthcare utilization, multivariable mixed-effects Poisson and negative binomial regression models were fit to examine changes over time, with time treated as both continuous and categorical (e.g., baseline, visits 2–4, and visits 5–7). Since the follow-up period was variable across participants, we included the length of total follow-up time or the time between visits as the exposure variable when appropriate.

Analysis of self-reported variables was limited to anyone with at least 2 or more visits to ensure that follow-up data were available (*n* = 123). For those program participants who sought care at UChicago Medicine, data were analyzed from the hospital administrative database to compare asthma-related ED visits and hospitalizations from 1 year before to 1 year after program participation. Hospital administrative data for healthcare utilization included anyone with at least 1 visit from CHW (*n* = 85). Analyses were performed using Stata 17 [27].

**Fig. 1:**
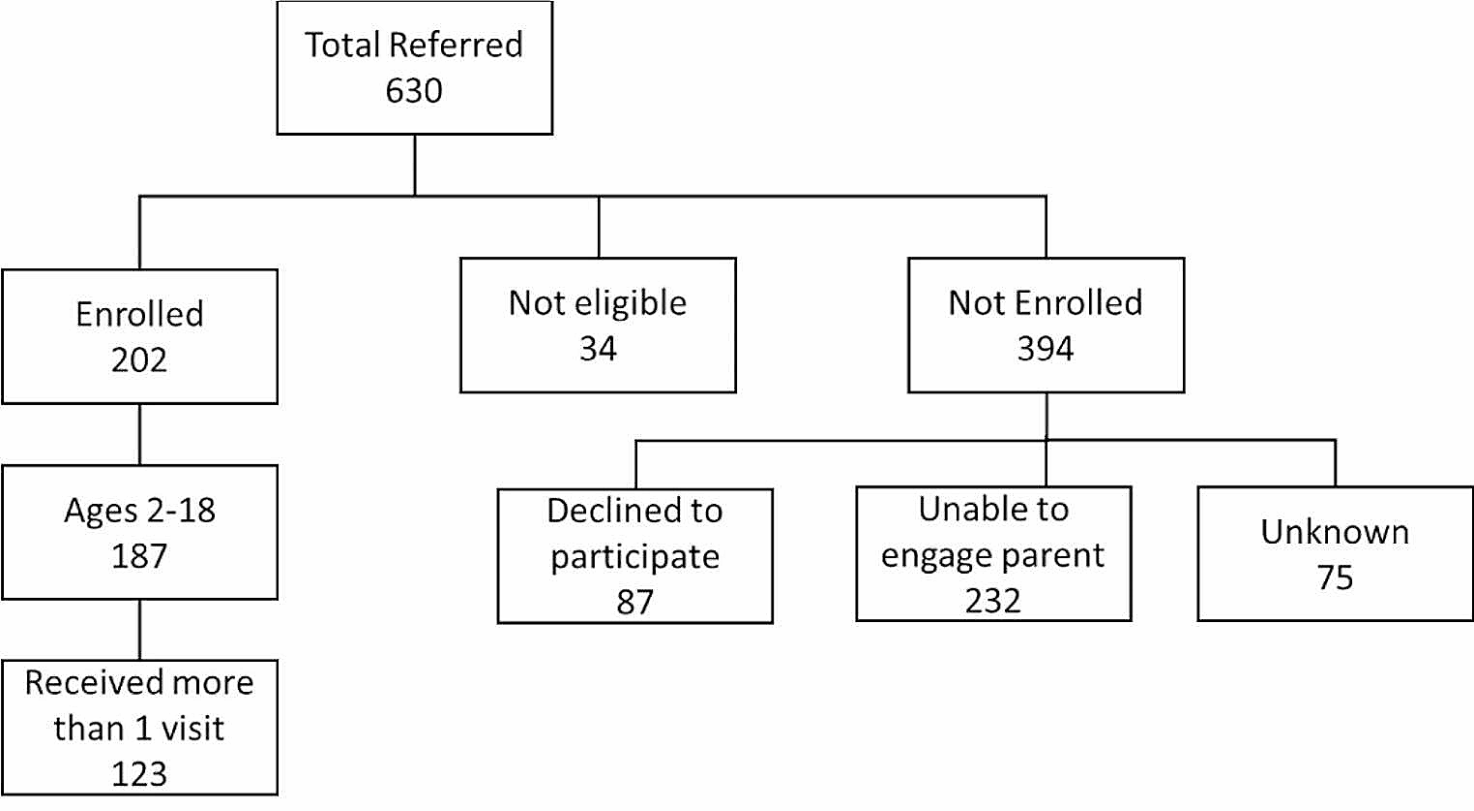
Summary of enrollment

## Results

During the study period, 630 patients were referred to the program; 202 (32.1%) were enrolled, 34 were not eligible (5.4%), and 394 (62.5%) were not enrolled in the program for various reasons as shown in Fig. 1. This analysis was limited to the 123 participants who received more than one visit from a CHW. Table 1 shows the baseline demographic and asthma characteristics of those who enrolled in the program and who received only one visit and those who received more than one visit from the program and had longitudinal self-reported data available.

The average age (±SD) of participants enrolled in the program was 8.8 (±4.4) years, 58.8% were male 89.3% were non-Hispanic Black, and 62.0% of participants had an annual household income of $21,000–30,000. A quarter (25.0%) of participants reported ever being admitted to the ICU and 32.1% reported hospitalization in the year before program participation. Over 40% (42.4%) of the participants had asthma that was partly controlled, 41.1% reported having and using their controller medicine every day, and 39.8% were exposed to tobacco smoke in the home. Reporting more hospitalizations in the year before program participation (*p* = 0.012) was associated with higher odds of dropping out after the first CHW visit.

CHWs provided 738 home/phone visits during the study period with participants receiving an average of 3.9 visits. Participants who did not drop out after their first home visit were in the program for an average of 177 days. On average, it took 42 days to convert a referral to the first program visit (Table 2).

**Table 1:** Demographic and baseline characteristics of children enrolled in the community health worker program and their association with dropout after the first visit (*n* = 187)

Participant Demographic and Baseline Characteristics	Participants who received at least one visit (*N* = 187)	Participants who received only one visit (*N* = 64)	Participants who received more than one visit (*N* = 123)	*P* value^a^
	n(% of sample)	n(% of sample)	n(% of sample)	
**Age at enrollment in years**				0.788
Mean (SD)	8.8 (4.4)	8.5 (4.8)	8.9 (4.2)	
2–5	53 (28.3)	19 (29.7)	34 (27.6)	
6–11	82 (43.9)	30 (46.9)	52 (42.3)	
12–18	52 (27.8)	15 (23.4)	37 (30.1)	
**Gender**				0.386
Male	110 (58.8)	35 (54.7)	75 (61.0)	
Female	77 (41.2)	29 (45.3)	48 (39.0)	
**Race/Ethnicity**				0.996
non-Hispanic Black	167 (89.3)	57 (89.1)	110 (89.4)	
Other	20 (10.7)	7 (10.9)	13 (10.6)	
**Household Income**				
≤ 20,000	52 (27.8)	16 (25.0)	36 (29.3)	
21,000–30,000	116 (62.0)	41 (64.1)	75 (61.0)	0.360
> 30,000	16 (8.6)	6 (9.4)	10 (8.1)	0.797
Unknown	3 (1.6)	1 (1.6)	2 (1.6)	0.897
**Referral Location**				
ED	72 (38.5)	24 (37.5)	48 (39.0)	0.217
Clinic	115 (61.5)	40 (62.5)	75 (61.0)	
**Asthma Control (** ***n*** ** = 177)**				
Well controlled	41 (23.2)	16 (26.7)	25 (21.4)	
Partly controlled	75 (42.4)	24 (40.0)	51 (43.6)	0.252
Uncontrolled	61 (34.5)	20 (33.3)	41 (35.0)	0.105
**Ever been admitted to the ICU**				
Yes	46 (25.0)	15 (24.2)	31 (25.4)	0.322
No	138 (75.0)	47 (75.8)	91 (74.6)	
**Tobacco smoke exposure in the home**				
Yes	74 (39.8)	27 (42.2)	47 (38.5)	0.375
No	112 (60.2)	37 (57.8)	75 (61.5)	
**Exposure to environmental triggers in the home**				NA
None	44 (26.3)	37 (57.8)	16 (13.1)	
1	48 (28.7)	27 (42.2)	31 (25.4)	
2	31 (18.6)	0	31 (25.4)	
3	22 (13.2)	0	22 (18.0)	
4–6	22 (13.2)	0	22 (18.0)	
**Availability and usage of controller medicine**				
Not available	64 (34.6)	19 (30.6)	45 (36.6)	
Available not using everyday	45 (24.3)	21 (33.9)	24 (19.5)	0.282
Available and using everyday	76 (41.1)	22 (35.5)	54 (43.9)	0.250
**Asthma knowledge score**				
≤ 5	34 (18.2)	10 (15.6)	24 (19.5)	
5.1–6	62 (33.2)	20 (31.2)	42 (34.2)	0.910
> 6	65 (34.8)	24 (37.5)	41 (33.3)	0.557
Unknown	26 (13.9)	10 (15.6)	16 (13.0)	0.412
**ER Visits reported in the 6 months before baseline**				
None	74 (39.6)	24 (37.5)	50 (40.6)	
≥ 1	113 (60.4)	40 (62.5)	73(59.3)	0.158
Mean (SD)	1.28 (1.82)	1.4 (2.28)	1.19 (1.53)	
**Hospitalization reported in the 12 months before baseline**				
None	127 (67.9)	38 (59.4)	89 (72.4)	
≥ 1	60 (32.1)	26 (40.6)	34 (27.6)	0.009*
Mean (SD)	0.53 (0.97)	0.80 (1.26)	0.39 (0.74)	

^a^*P*-values from a logistic regression model for attrition

* *p*-value significant

NA – exposure to environmental triggers was not included in the attrition model because detailed information on triggers was not available for those that only received one visit. The variable is included in this table because it is used subsequently in the regression models

**Table 2:** Program Participation (*n* = 187)

	Participants who received only one visit (*N* = 64)	Participants who received more than one visit (*N* = 123)
	Mean (SD)	Mean (SD)
Total number of visits per (home and phone) participant	1	5.5 (2.3)
Total home visits per participant	1	3.3 (0.96)
Total phone visits per participant	0	2.2 (1.39)
Number of days elapsed between enrollment and first program visit	49.1 (52.9) Median: 29	38.6 (37.2) Median: 28.5
Average number of days in the program	NA	177.6 (142.2)
Total number of visits provided by the program	738	
Home visits	471	
Phone Visits	267	

### Asthma Morbidity

**Fig. 2:**
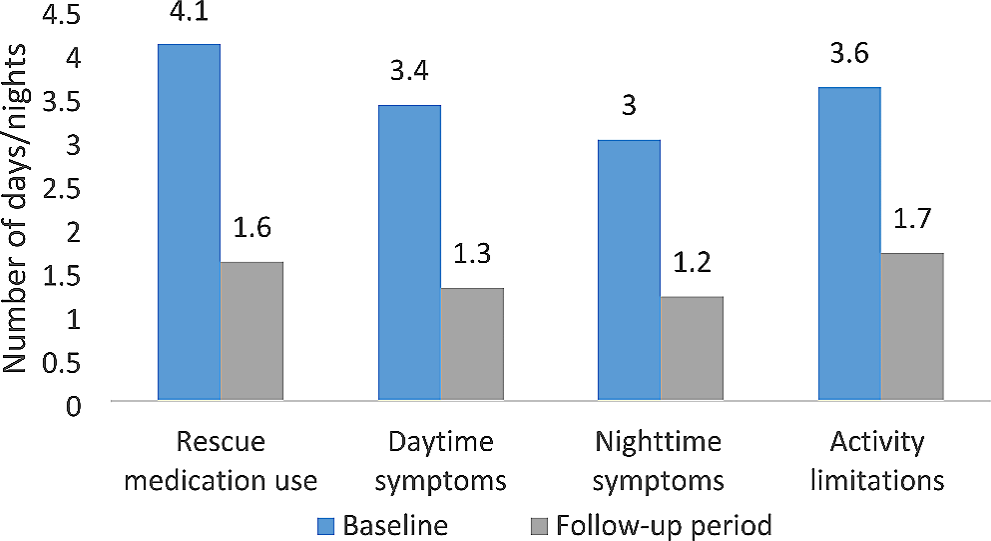
Symptom days/nights, rescue medication usage, and activity limitations in the past 2 weeks reported at baseline versus median reported across all follow-up visits (all *p* < 0.001 from paired t-test) (*n* = 119)

There were significant reductions in the average number of days and nights of symptoms, rescue medication usage and activity limitations reported in the past two weeks at baseline compared to the median reported across all follow-up visits (all *p* < 0.001; Fig. 2). The average duration of daytime symptoms decreased from 4.1 days at baseline to 1.6 at follow-up while activity limitations decreased from 3.6 days at baseline to 1.7 days at follow-up.

Multivariable mixed-effects negative binomial regression models for the number of days of symptoms, rescue medication usage and activity limitation (controlling for demographic variables, enrolled in the ED vs. not, asthma control, use and availability of controller medicine, history of ICU visits, total knowledge score, number of triggers present and season) showed that there was a significant decrease in all of these outcomes between baseline and after program visits with a greater decrease among those receiving more visits (Table 3). Rescue medication usage decreased from 4.3 days at baseline to 2.2 days after 2–4 visits and to 1 day after 5–7 visits (*p* < 0.001), which was a 49% and 76% decrease, respectively. After receiving 2–4 CHW visits, there was a 59% decrease, and after 5–7 CHW visits there was an 80% decrease in nighttime symptoms compared to baseline. Similar results were obtained with a multivariable regression model in which time was treated as a continuous variable (results not shown).

**Table 3:** Mixed effects negative binomial regression models for rescue medication usage, symptom frequency, and activity limitations in the past 2 weeks (*n* = 115)

OUTCOME	Visit	Predicted Mean number of days	IRR	95% CI	*P* value
Rescue medication use	BL	4.3	Reference		
	2-4	2.2	0.51	0.36–0.72	< 0.001
	5-7	1.0	0.24	0.16–0.37	< 0.001
Daytime symptoms	BL	3.8	Reference		
	2-4	1.7	0.44	0.32–0.61	< 0.001
	5-7	1.0	0.26	0.17–0.38	< 0.001
Nighttime symptoms	BL	4.0	Reference		
	2-4	1.6	0.41	0.28–0.60	< 0.001
	5-7	0.78	0.20	0.12–0.33	< 0.001
Activity limitations	BL	4.4	Reference		
	2-4	2.1	0.48	0.33–0.68	< 0.001
	5-7	1.3	0.29	0.18–0.45	< 0.001

^a^ Adjusted for baseline age, gender, race, income group, enrolled in the ED vs. not, asthma control level, availability and usage of controller medication, asthma severity (history of ICU visits due to asthma at time of enrollment), knowledge score (categorical), number of triggers present, and season

IRR = Incidence-rate ratio; CI = Confidence Interval; BL = Baseline

### ED Visits, Hospitalizations and Missed Work and School Days (Self-Reported)

Multivariable mixed-effects regression models demonstrated a significant decrease in ED visits and missed school and work days during the intervention period compared to 6 months before program participation (Table 4). There was a 70% decrease in ED visits and a 72–73% decrease in missed school days and missed work days during the intervention period compared to the 6 months before program participation (all *p* < 0.001). No significant difference was found in hospital admissions during the intervention period compared to the 1 year before program participation (*p* = 0.14). Similar results were observed when examining the dose of the program intervention compared to baseline, with early and late effects both being significant (all *p* ≤ 0.001) for ED visits, missed school days and missed work days (Table 5). After receiving 2–4 CHW visits, there was a 58% reduction, and after 5–7 CHW visits, there was an 86% reduction in ED visits compared to baseline. Similarly, after receiving 2–4 CHW visits, there was an 82% reduction in missed school days for children compared to baseline.

**Table 4:** Mixed-effects Poisson or negative binomial^a^ regression model for self-reported ED visits, hospitalizations, missed school days, and missed work days looking at baseline vs. intervention period^b^ (*n* = 115)

OUTCOME	Time	Predicted Mean Number of Events	IRR	95% CI	*P* value
ED visits	BL	1.19	Reference		< 0.001
	Intervention	0.36	0.30	0.21–0.43	
Hospital admits	BL	0.30	Reference		0.14
	Intervention	0.19	0.63	0.35–1.16	
Missed school days	BL	5.6	Reference		< 0.001
	Intervention	1.5	0.28	0.17–0.44	
Missed work days	BL	4.3	Reference		< 0.001
	Intervention	1.2	0.28	0.14–0.55	

^a^ Negative binomial models were used for missed school and work day outcomes, while Poisson models were used for ED visits and hospitalizations

^b^ Adjusted for baseline age, gender, race, income group, enrolled in the ED vs. not, asthma control level, availability and usage of controller medication, asthma severity (history of ICU visits due to asthma at time of enrollment), knowledge score (categorical), number of triggers present, and season

IRR = Incidence-rate ratio; CI = Confidence Interval; BL = Baseline

**Table 5:** Mixed-effects Poisson or negative binomial^a^ regression model for self-reported ED visits, hospitalizations, missed school days, and missed workdays looking at baseline vs. multiple time points^b^ (*n* = 115)

OUTCOME	Visit	Predicted Mean Number of Events	IRR	95% CI	*P* value
ED visits	BL	0.47	Reference		
	2-4	0.20	0.42	0.28–0.65	< 0.001
	5-7	0.07	0.14	0.07–0.29	< 0.001
Hospital admits	BL	0.12	Reference		
	2-4	0.09	0.78	0.39–1.57	0.48
	5-7	0.05	0.4	0.14–1.15	0.08
Missed school days	BL	8.0	Reference		
	2-4	1.4	0.18	0.09–0.36	< 0.001
	5-7	1.1	0.13	0.05–0.37	< 0.001
Missed work days	BL	11.0	Reference		
	2-4	1.3	0.11	0.04–0.30	< 0.001
	5-7	1.1	0.1	0.03–0.37	0.001

^a^ Negative binomial models were used for missed school/work days outcomes, while Poisson models were used for ED visits and hospitalizations

^b^ Adjusted for baseline age, gender, race, income group, enrolled in the ED vs. not, asthma control level, availability and usage of controller medication, asthma severity (history of ICU visits due to asthma at time of enrollment), knowledge score (categorical), number of triggers present, and season

IRR = Incidence-rate ratio; CI = Confidence Interval; BL = Baseline

### ED Visits and Hospitalizations (Administrative Database)

**Fig. 3:**
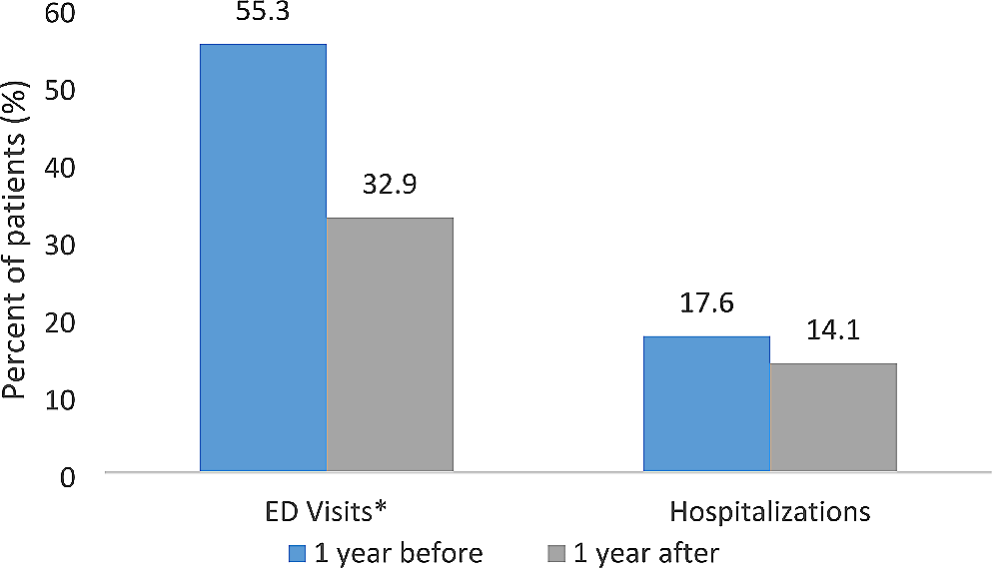
Emergency department visits and hospitalizations (≥ 1) one year before program participation and one year after program participation (*n* = 85) *Statistically significant (*p* < 0.004) according to McNemar’s test

There was a significant difference in the proportion of patients with any ED visits one year before program participation compared to one year after program participation (55.3% vs. 32.9%; *p* < 0.001), while no significant difference was found in hospitalizations (Fig. 3). Similar results were obtained for follow-up vs. baseline comparison in a multivariable mixed-effects Poisson regression model adjusting for demographic and asthma characteristics (Table 6). The average number of ED visits was 0.92 one year before and 0.44 one year after program participation, corresponding to a 52% decrease (*p* < 0.001).

**Table 6:** Mixed-effects Poisson regression^a^ models for ED visits and hospitalizations reported 1 year before and 1 year after program participation (*n* = 79)

OUTCOME	Time	Predicted Mean number of events	IRR	95% CI	*P* value
ED visits	1 year before	0.92	Reference		
	1 year after	0.44	0.48	0.32–0.72	< 0.001
Hospitalizations	1 year before	0.27	Reference		
	1 year after	0.18	0.67	0.34–1.31	0.24

## Discussion

The SSPAC CHW program included warm-hand off of patients from specialty asthma clinics to a CHW, followed by home visits where tailored asthma management education was provided. The CHWs worked closely with the patient’s providers to implement comprehensive care coordination through a range of interventions, from helping to set up regular appointments, ensuring that prescribed medications are filled, regularly assessing medication adherence, and addressing household triggers and their remediation. The CHWs essentially functioned as health advocates for the families they served. Additionally, the program was implemented with flexibility to adapt to each family’s situation and based on the family’s needs, the number of visits and duration of participation varied.

An evaluation of the SSPAC’s CHW home visit program that was integrated across multiple hospitals and health centers, demonstrated a significant reduction in both short-term and long-term outcomes of interest for asthma patients enrolled in the program. Specifically, day-time and night-time asthma symptoms, rescue medication usage, and activity limitations reported in the past two weeks decreased significantly after program participation. There were also significant changes in the long-term outcomes of asthma related ED visits, missed school and workdays after program participation decreased compared to those at baseline. Other long-term outcomes, including hospitalization, did not see a significant change.

The results of this evaluation are similar to those of prior research studies that have shown that CHW models can be effective at reducing healthcare utilization, decreasing costs, and improving asthma morbidity [10, 13, 21]. While previous studies had fixed follow-up periods and results are available only for overall program effectiveness, this evaluation provides new insights into the number of CHW visits needed to observe changes in health outcomes. The early effects of this program and visible reductions in asthma morbidity and healthcare utilization after just 2–4 visits from a CHW suggest that despite the difficulty with participant retention, the program could still be effective with just a few interaction points. However, the magnitude of change in outcomes after 5–7 visits suggested that long-term follow-up may also be needed in QI programs. These findings also suggest that a tailored approach based on patient needs is warranted instead of a one-size –fits-all model that dictates a set number of visits for every enrolled patient.

The results shared here demonstrate the effects of a community health worker model in which CHWs are part of the care team, where they can build patient relationships and be seen and accepted as a part of the patient’s continuum of care. Furthermore, the recruitment of community health workers from targeted communities who are culturally competent and can relate to families is recommended and effective, especially if this is done in conjunction with adequate certifications that take their years of experience into account [7].

While other similar programs have demonstrated reductions in hospitalizations after the implementation of CHW models [13, 21], significant reductions in hospitalizations according to self-reported or administrative data were not observed. This could be the result of those participants with greater number of hospitalizations dropping out after the first visit and leaving those with less severe asthma in the program. Similarly, hospitalizations were minimal in the administrative database, with data access limited to one institution within the SSPAC. Thus, we were not able to capture healthcare utilization from partner hospitals to formulate further insights.

This study is limited by the fact that it is an evaluation of a quality improvement program and not a randomized controlled trial. Although attempts were made to identify a retrospective control group for comparison with those receiving the program intervention, data limitations to UCM patients led to a very small sample size. Utilizing data from multiple hospitals or using insurance claims data with a larger sample size may be more helpful in future analyses to evaluate health care utilization and cost effectiveness. Self-reported data from parents/guardians may be affected by recall and social desirability biases; however, data collected through self-reports and hospital administrative data showed similar results for health care utilization. Finally, there was considerable dropout of participants over time, which could bias these results and demonstrate the true difficulties faced in real-world programs.

A cost-effectiveness analysis would provide additional support to this program evaluation study. Further analysis examining the longer-term effects of the CHW program at 2, 3, or 4 years of follow-up would be beneficial. The SSPAC program required adaptation to a fully virtual model during the COVID-19 pandemic, and healthcare utilization patterns had already changed across the US during this time period. Therefore, the analysis was limited to only one year of follow-up from the administrative database. Further studies examining program outcomes among patients receiving program services during the pandemic would be valuable to add to the literature.

In conclusion, it was beneficial to implement a CHW home visit program across multiple institutions as standard practice to reduce asthma disparities on the South Side of Chicago. The findings demonstrated a reduction in asthma morbidity and asthma-related ED visits, missed school days and missed work days. Overall, our findings show that a collaborative effort across health centers and hospitals to integrate a CHW home visiting program as a standard of care for pediatric asthma patients has the potential to reduce asthma disparities for under resourced communities in urban settings. The results contribute to support of the CHW role as part of the clinical care team and substantiate reimbursement from health plan providers.

## Data Availability

The data used in this evaluation are protected by HIPAA and sharing of individual study data with those outside the study team is prohibited by our institution.
